# Prevalence of anemia and its associated factors among children aged 6–23 months, in Ethiopia: a systematic review and meta analysis

**DOI:** 10.1186/s12889-023-17330-y

**Published:** 2023-12-02

**Authors:** Molla Azmeraw, Amare Kassaw, Samuel Derbie Habtegiorgis, Agimasie Tigabu, Abraham Tsedalu Amare, Kindie Mekuria, Dessie Temesgen, Alemu Birara Zemariam, Gashaw Kerebeh, Berihun Bantie, Dessie Alemnew, Biruk Beletew Abate

**Affiliations:** 1 Department of Nursing, College of Health Science, Woldia University, P.O. Box: 400, Weldiya, Ethiopia; 2 Department of Epidemiology, College of Medicine and Health Science, Debre Markos University, Debre Markos, Ethiopia; 3 Department of Comprehensive Nursing, College of Health Science, Debre Tabor University, Debra Tabor, Ethiopia; 4 Department of Pediatrics and Child Health Nursing, College of Health Science, Debre Tabor University, Debra Tabor, Ethiopia; 5 Department of Nursing, Faculty of Health Science, Debark University, Debark, Ethiopia; 6 Pediatics and child health nursing, Woldia University, Woldia, Ethiopia

**Keywords:** Anemia, children under two years, Ethiopia

## Abstract

**Introduction:**

Despite several strategies exist for anemia prevention and control, it has been the major public health important problem in the world. Numerous immediate and long-term health issues were reported in children who have history of anemia including decreased work productivity in adult hood period. Although analyzing data on burden and risk factors of anemia are the recommended action areas of World Health Organization framework for accelerating anemia reduction, the aggregated national burden and contributors of anemia in Ethiopia has not been determined so far. There for, this systematic and meta-analysis study is aimed to assess the pooled prevalence and associated factors of anemia among children aged 6–23 months in Ethiopia.

**Methods:**

The electronic databases including PubMed, Scopus, EMBASE, Web of Science, Science Direct, Google scholar and institutional repositories were searched using search terms. The studies that reported the prevalence and/or risk factors of anemia in children 6–23 months of age were included. The JBI quality assessment tool was used to evaluate the quality of each study. The data was extracted with Microsoft Excel, 2019 and analyzed with STATA 17.0 statistical software. A random effect model was used to estimate the pooled prevalence of anemia and its associated factors. The Cochrane Q-test statistics and I^2^ test were used to measure heterogeneity between the included studies. Furthermore, publication bias was examined using the funnel plot graph and statistical tests (Egger’s and begg tests). Outliers also visualized using Galbraith plot. When necessary, sensitivity analysis was also employed to detect small study effect.

**Result:**

Ten studies with a total population of 14, 733 were included for analysis. The pooled prevalence of anemia among children aged 6–23 months of age in Ethiopia was found to be 57.76% (95%CI; 51.61–63.91; I^2^ = 97.192%; p < 0.001). Having history of diarrhea AOR = 2.44 (95%CI: 1.03–3.85), being stunted AOR = 2.00 (95%CI: 1.38–2.61), living in food insecure house hold AOR = 2.08 (95%CI: 1.10–3.07), consuming less diversified food AOR = 2.73 (95%CI: 2.06–3.39) and being 6–11 months of age AOR = 1.59 (95%CI: 1.23–1.95) were associated with anemia.

**Conclusion and recommendation:**

The prevalence of anemia is in the range of severe public health problem among children aged 6–23 months in Ethiopia. Diarrhea, stunting, house hold food insecurity, dietary diversity, and age were the predictors of anemia. Further, prospective cohort and random controlled trial studies are recommended. Further, random controlled trial especially effectiveness of nutritional education interventions trial is important. To reduce prevalence of anemia, strengthening diarrhea reduction program, securing household food insecurity, preventing stunting, giving special attention for infants age 6–11 months and encouraging food diversification are important.

**Supplementary Information:**

The online version contains supplementary material available at 10.1186/s12889-023-17330-y.

## Introduction

Anemia is a strong indicator of overall health and defined as a condition in which the number of red blood cells or the hemoglobin concentration within them is lower than normal and insufficient to meet an individual’s physiological needs [1, 2]. It is one of the public health important problems in both developed and developing nations [3]. World Health Organization (WHO) considered anemia as mild, moderate and severe public health problem when its prevalence is 5-19.9%, 20-39.9% and 40% of the population, respectively [2].

There is geographical difference in anemia distribution. Africa, South-East Asia region, and Eastern Mediterranean region were the major contributor regions of anemia in the globe. Regarding to its burden, 27% of the world’s population were anemic in 2013 [4]. Women in reproductive age and children 6–59 months were the major contributors of anemia in the general population [2, 5]. Another global study on anemia predicted that 40% of all children aged 6–59 months were anemic in 2019 which was 48% in 2000 and 42.6% in 2011 [5, 6]. Anemia accounts 89% of all disability in developing countries [4]. Based on anemia severity 21%, 18% and 1% of children aged 6–59 months had mild, moderate, and severe anemia, respectively in 2019 [7]. A previous study report also showed that the burden is highest in Central and Western Sub-Saharan Africa(SSA) [4]. In East Africa, the prevalence of anemia was reported as 53% in 2019 [7]. According to the global burden disease report 8.2 million, 10 million, 1.02 million in 1990 and 12.35 million, 14.39 million, 1.28 million in 2013 children were with mild, moderate, and severe anemia, respectively in Ethiopia [4]. The Ethiopian Demographic and Health Survey (EDHS) report also described that 57% of the children under the age of 5 years were anemic in 2016 [8]. Anemia levels were highest in those aged 6–23 months, with 72% of these children having anemia [8]. Even though iron‐deficiency remains the dominant cause of anemia in all populations33%, 40% and 42% of non-pregnant women, pregnant women, and children were affected by anemia worldwide, respectively [4]. Hemoglobinopathies, infections, chronic kidney diseases, gastrointestinal disorders, and gynecological issues are also the other more significant causes of anemia [4, 9]. World Health Organization considered, the cause of anemia in area where prevalence of anemia greater than 40% were multifactorial, no single cause responsible for anemia [10]. During early childhood period, iron needs due to rapid growth, inadequate iron intake due to exclusively breastfed without iron supplementation, inadequate dietary iron intake, prematurity (low iron store), low availability of dietary sources of iron secondary to low socioeconomic status and dietary restrictions were the possible causes of anemia [11, 12].Despite inconsistency among existing studies, several factors were associated with high prevalence of anemia among children aged 6–23 months in Ethiopia. Age, food insecurity, poor dietary diversity, time of complementary feeding initiation (late or early), diarrhea, cow milk initiation before one-year, poor breast-feeding practice, having poor wealth index, maternal age and educational status were the predictors of anemia in this age group [13–20].

Numerous immediate and long-term health issues affect children who have or have history of anemia. In anemic children, the oxygen-carrying capacity of blood is low which leads to lack of oxygen supply to vital organs including the fast-growing children’s brain and heart [3, 11]. Anemia negatively affects mental, motor, and cognitive development, which in turn lead to social withdrawal, attention deficit, poor growth, and impaired school performance (learning difficulties) of children which may be irreversible, especially in children less than 2 years, despite adequate therapy [11, 21]. In severe cases, anemia in children is ends with heart failure and death [11, 22]. the harmful impact of anemia is not only to young children but also it hits women who are pregnant or planning a pregnancy [4]. Anemia has also a tendency to hinder behavioral development and raising the likelihood of illness including high risk of infection [3, 21, 22]. Anemia can also reduce work productivity later in life [3, 23]. In the long run, children who were anemic during infancy had a discrepancy of 5–10 points on intelligence tests and other cognitive performance examinations in school than children who were not anemic.

Several strategies exist for anemia prevention and control. These include improvement of dietary intake, food diversification, food fortification, supplementation with iron and other micronutrients, appropriate infectious disease prevention and control, delayed cord clamping and health education [23]. Optimization of maternal nutritional status can also decrease inter-generational iron depletion anemia during infancy [3]. Despite implementation of several strategies, a significant number of children have been living with anemia.

Although high proportion of anemia reported in infants and preschool children in the previous global study [24], the burden of anemia among children less than 2 years has not been determined in Ethiopia. In addition, analyzing data on cause and risk factors of anemia is one of the action areas of WHO framework for accelerating anemia reduction. Therefore, having knowledge about the pooled prevalence and risk factors of anemia in the population (children 6–23 months) helps to decide on appropriate interventions, resource mobilization and monitor and evaluate the impact and safety of public health programs. It may also contribute to the national strategy for child health and wellbeing. Furthermore, it informs policy by providing information to health practitioners, policy makers and other stake holders that will provide appropriate measures to improve the health services particularly to childhood anemia in the study area.

## Research question

What is the national burden of anemia among children aged 6–23 months in Ethiopia?

What are the risk factors of anemia among children aged 6–23 months in Ethiopia?

### Objective

To perform a systematic review of available research literature regarding the prevalence and associated factors of anemia among children aged 6–23 months in Ethiopia, 2023.

### Specific objective

To estimate the pooled prevalence of anemia among children aged 6–23 months in Ethiopia.

To determine the risk factors that exposed children aged 6–23 months for anemia in Ethiopia.

## Methods

### Study design and setting

All the studies which have been conducted on anemia among children aged 6–23 months in Ethiopia were cross-sectional by study design. There is inconsistency in prevalence report and controversies in reporting the risk factors that exposed children for anemia. In addition, cross-sectional studies are lower in evidence generation than systematic review. Systematic review and meta-analysis are driven by evidence-based medicine movement and Cochrane collaboration. They are advantageous in reducing bias, replicability, in resolving controversies, and in providing reliable basis for decision makings than cross-sectional studies. Therefore, in this systematic review and meta-analysis, the authors reviewed studies conducted on anemia among children aged 6–23 months in Ethiopia to estimate the pooled prevalence of anemia and to resolve the existing controversies between studies. Cochrane Handbook for Systematic Reviews of Interventions recommends at least 10 studies for publication bias assessment. Only two studies may be enough to do a meta-analysis study. Cochrane Handbook for Systematic Reviews of Interventions also recommends at least two studies to run forest plot [25]. The Preferred Reporting Items for Systematic Review and Meta-analyses (PRISMA) guideline was used to review the existing article about anemia in the population described [26]. Ethiopia is one of the east African countries situated in the horn of Africa having a total population of 117.88 million, 58.98 million males and 58.90 million females in 2021 [27]. By age distribution, nearly half of Ethiopians are under age 15 (47%), while 4% are age 65 and older [8]. In 2021, Ethiopia will have 3.65 million births. That is 10,014 per day, ranking eighth. accounting for 22.17% of the total population. In terms of urbanization, it is also placed 219th with the urban population of 25.59 million [27]. Administratively, it has 10 regions and two administrative cities [8].

### Searching strategy

The presence of systematic review protocol on the topic of prevalence and associated factors of anemia among children aged 6–23 months in Ethiopia was checked via searching different databases including PubMed, Scopus, EMBASE, Web of Science, ScienceDirect, Google scholar, the national health center review and institutional dissemination databases and Prospero for systematic review. As far as our search strategy is concerned, there was no systematic review conducted on our topic of interest. Thus, the actual search was conducted from March 2, 2023 to May 14, 2023. In order to minimize time-lag bias, the searching process was updated on June 15, 2023. Our search strategy focused on all published and unpublished studies with epidemiological data on the prevalence and associated factors of anemia among children aged 6–23 months in Ethiopia without restriction for publication year. key terms: children, anemia and Ethiopia with combinations of MeSH terms were used as searching strategies. These key terms were combined using Boolean operators “AND/OR’’ to narrow the search in the databases. The searching using Boolean operators in PubMed was(“Prevalence OR Frequency OR proportion”) AND (“Associated Factors” OR “determinants” OR “risk factors” OR “causes”) AND (“anemia” OR “anaemia”) AND (“child**”OR “pediatric**”OR “paediatric**”) AND “6–23 month**”) AND “Ethiopia” and Science direct and google scholar “Prevalence ”AND (“Associated Factors” OR “determinants”) AND “anemia” AND “children”AND “6–23 month”) AND “Ethiopia”were used. In addition, the electronic searching strategy was supplemented by manual searches to identify relevant unpublished studies. Search terms used included “Anemia”, “young children” or “infant” or “children aged 6-23month”,” and “Ethiopia.” The reference lists of included studies were also screened for the presence of additional studies. Three authors (MA, DT and AK) conducted the searching process. References were managed using End Note software version X8. This systematic review and meta-analysis study was not registered with PROSPERO.

### Study selection and eligibility

The three authors independently and meticulously appraised the contents of each of the identified article (B.B.A., A.T.A. and A.T.). The retrievals were appraised for inclusion in the final review by using their titles and abstracts. Finally, the appraisal process completed by reviewing full text of the papers. Those studies which met the following inclusion criteria were considered to be included in the study.

### Inclusion criteria

#### Population

Studies carried out in children aged 6–23 months.

#### Study design

All observational studies (all article or publication reporting prevalence of anemia and/or associated factors were considered for inclusion).

#### Study area

Those studies conducted only in Ethiopia.

#### Study design and outcome definition

Original studies and government surveys, EDHS which reported at least the prevalence and/or one associated factors of anemia among children aged 6–23 months in Ethiopia were included. Studies defined anemia as hemoglobin levels of < 11 g/dl, and for duplicate publications, only the most recent, comprehensive publication with the largest sample size were included.

#### Language

only studies reported in English language were included.

Publication year- all articles published before June 23,2023 were included.

### Exclusion criteria

**Fig. 1:**
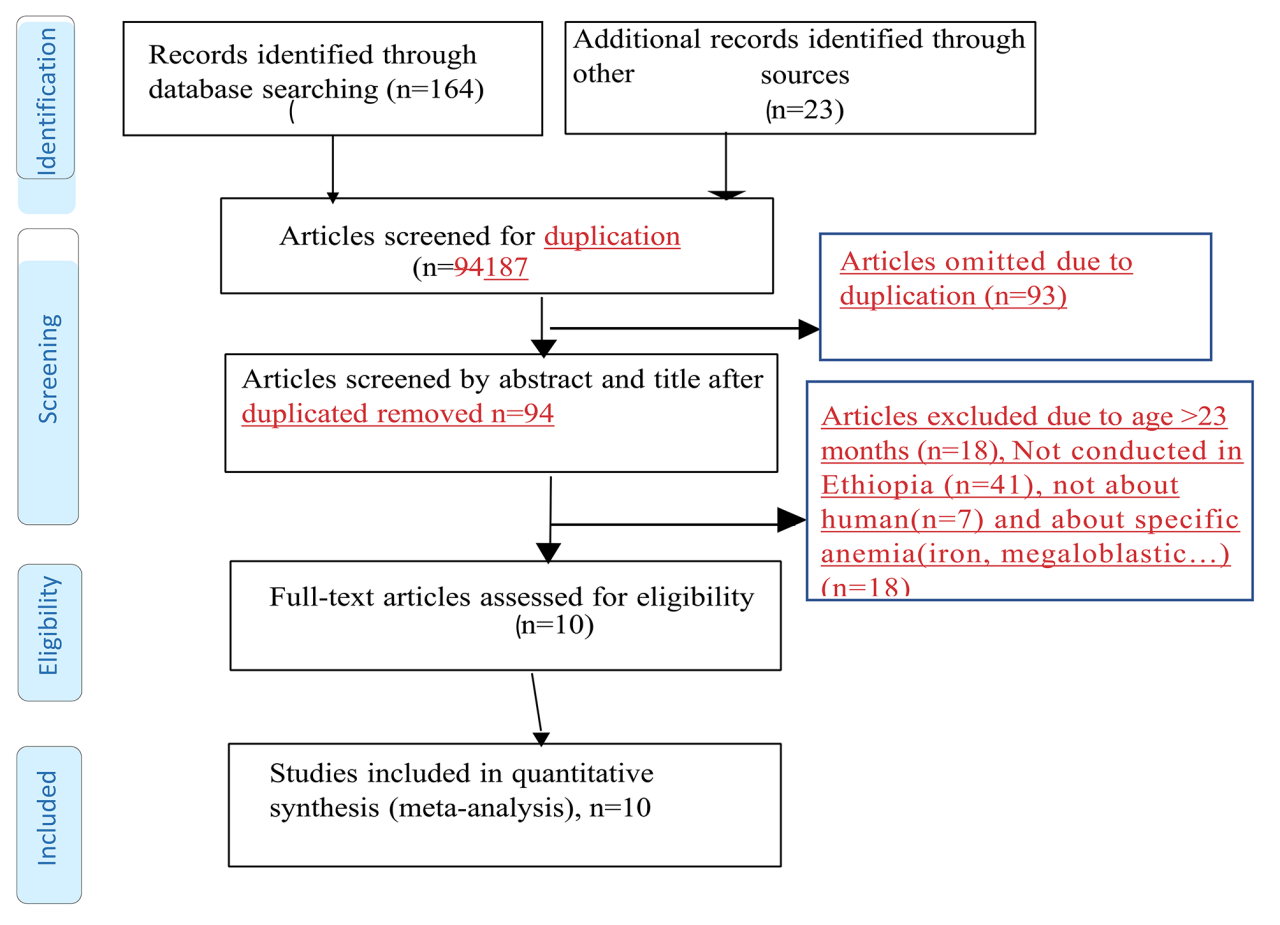
PRISMA flow diagram showing the selection of studies for a systematic review and meta-analysis, 2023

The four reviewers (S.D.H, B.B., K.M. and A.B) performed the selection of the studies independently and blindly after thorough screening of the abstracts and the full texts of the studies. Methodologically flawed articles were excluded. Any differences arose throughout the review process were settled by consensus, and if they persisted despite debate, a third party was consulted to resolve the issue. Review studies, commentaries were also excluded. In addition, studies conducted in a population with hemoglobinopathies like sickle cell anemia and with no information on the tools used to diagnose anemia were also excluded. The processes of identifying, screening and including or excluding records were done using the PRISMA flow diagram guideline (Fig. 1).

### Publication condition

Studies which meet the eligibility criteria were included regardless of their publication year and publication status (published, unpublished and grey literature, etc.)

### Quality assessment

The quality of the included studies were independently assessed by three authors (A.K., M.A., and G.K.) using the Joanna Briggs Institute (JBI) critical appraisal tool for cross-sectional studies. We categorized the overall quality scores as low quality (high risk of bias), moderate quality (moderate risk of bias), and high quality (low risk of bias) for scores of < 50, 50–75, and > 75%, respectively [28]. The appraisal tool has the following 8 criteria: (1) inclusion criteria, (2) description of study subject and setting, (3) valid and reliable measurement of exposure, (4) objective and standard criteria used, (5) identification of confounder, (6) strategies to handle confounder, (7) outcome measurement, and (8) appropriate statistical analysis. Studies were considered low risk whenever fitted to 50% and/or above in the quality assessment checklist criteria [28, 29].

## Outcome

The primary outcome variable of this review was prevalence of anemia. The outcome was measured based on the proportion of children aged 6–23 months with their hemoglobin level less than 11 g/dl. The prevalence of anemia reported in different studies were presented by pooling the prevalence reported in each included article. To take the study-specific true effects across the included studies into consideration, the random effect meta-analysis model was employed. A random effects model for the reported proportion was used to present the pooled prevalence of anemia. Furthermore, different factors that are reported which affect the prevalence of anemia were also identified. These factors were presented using a narrative synthesis through generation of different themes with their pooled effect size (adjusted Odds Ratio, AOR).

### Data analysis

The pooled prevalence of anemia with 95% confidence intervals (CIs) was calculated using a random-effects model. In addition, the Cochran’s Q test was utilized to assess the significance of the heterogeneity (p≤0.05 was considered as statistically significant). I -Squared (I^2^) values of < 25%, 25–50%, 50.01-75% and > 75% were interpreted as a low, medium, and high heterogeneity, respectively. To identify publication bias, funnel plots were created and then applied Egger’s test. Publication bias was declared when the Egger’s and begg test became significant or p-value<0.05. Next, a Galbraith plot was formed to locate any studies that had statistical outliers.

### Result

#### Study selection

A total of 187 studies were identified using electronic searches (through Databases searching (n = 187)) that were conducted up to June, 2023. Of the articles accessed, the duplicated studies were handled using both End Note software version X8 reference manager software and manual comparison of titles/abstracts. After combining all reference from all data bases, using ‘find duplicates’ from End Note software version X8 “reference” menu bar, the duplicated references were removed. After duplication removed, a total of 94 articles remained (93 duplicated). Then, 94 studies were screened using their title and abstract for full-text review. Of the articles screened, 18 were excluded because they are not our population interest (their study extended to children age > 23 month), not conducted in Ethiopia (n = 41), not about human(n = 7) and about specific anemia (iron, megaloblastic…) (n = 18). The rest 10 articles were checked for their quality. Fortunately, all 10 articles with (n = 14, 733 study participants) fulfil the quality assessment (no article left due to quality assessment). The authors considered them for the prevalence and/ or associated factors analysis (Fig. 1).

### Characteristics of the studies

Out of 10 eligible studies two of them were conducted in similar population and season [14, 15]. Among the two studies, the one which has small sample size, Gebrehaweria Gebremeskel M et al [14] was not considered in the analysis. The characteristics of included studies were described in Table 1 [13–20]. Four studies were Ethiopia Demographic Health Survey [14, 15], 2 in Amhara [17, 20], 3 in Oromia [18, 19, 30] and 3 in SNNPE [13, 16, 31]. Of the total population 14, 733, the major contributor of the population was EDHS (9878) followed by 1992 from SNNPE, 1920 from Oromia and 943 from Amhara. All studies were cross-sectional in study design and 1 institutional and 9 community-based studies by setting. The studies were published from 2015 to 2022. The number of participants included in the studies, ranging from 216 [30] to 2970 [15] (Table 1). The population age distribution of the included studies was similar, ranged from 6 to 23 months. Although, Woldie, H. et al [20], Sorsa, Aet al [18] and Heinrichs, H. et al [15] did not report the mean age, it ranged from 11.58 ± 2.75 [19] to 14.7 ± 5.1 months [17]. The reported prevalence of anemia ranged from 44.4% [18] up to 72.3% [14] across the included studies (Table 1). The discrepancy between number of eligible study and number of studies listed in the table (Table 1) is due to one study report. From which three rounds of EDHS report which are in one article were considered for analysis [15].

**Table 1:** Characteristics of included studies conducted on prevalence of anemia in children aged 6–23 months in Ethiopia, 2023

Authors name	Setting	Region	Design	Sample size	N. of outcome	MA ± SD in M	Prevalence (%)	Quality
Alemayehu, M. et al., 2019 [13]	CB	SNNPE	CS	993	650	14.96 ± 5.4	65.7	5
Woldie, H. et al., 2015 [20]	HI	Amhara	CS	366	231	NR	66.6	5
Tegegne, M. et al., 2022 [19]	CB	Oromia	CS	787	369	11⋅58 ± 2⋅7	47.9	7
Gebrehaweria Gebremeskel M et al., 2020 [14]^#^	CB	EDHS	CS	2554	1847	13 ± 4	72.3	5
Sorsa, A. et al., 2021 [18]	CB	Oromia	CS	917	407	*NR*	44.4	6
Molla, A. et al., 2020 [17]	CB	Amhara	CS	577	252	14.7 ± 5.1	47.5	7
Malako, B.G. et al., 2018 [16]	CB	SNNPE	CS	522	255	13.65 ± 5.4	52.6	5
Malako BG et al., 2019 [31]	CB	SNNPE	CS	477	248	13.69 ± 5.41	52	6
Roba KT et al., 2016 [30]	CB	Oromia	CS	216	116	13.35 ± 4.8	53.7	6
Heinrichs, H. et al., 2020 [15]*	CB	EDHS	CS	1,290	918	*NR*	71.2	6
Heinrichs, H. et al., 2020 [15]**	CB	EDHS	CS	2970	1809	*NR*	60.9	6
Heinrichs, H. et al., 2020 [15]***#	CB	EDHS	CS	3064	2204	*NR*	71.9	6

N.B: -CB-Community-based, CS-Cross-sectional, HI-health institution,, M-Month, MA-Mean Age, NR-Not Reported, SD-Standard Deviation, SNNPE-South Nation Nationality and Peoples of Ethiopia, * EDHS 2005, ** EDHS 2011, *** EDHS 2016, # similar studies

### Prevalence of anemia

**Fig. 2:**
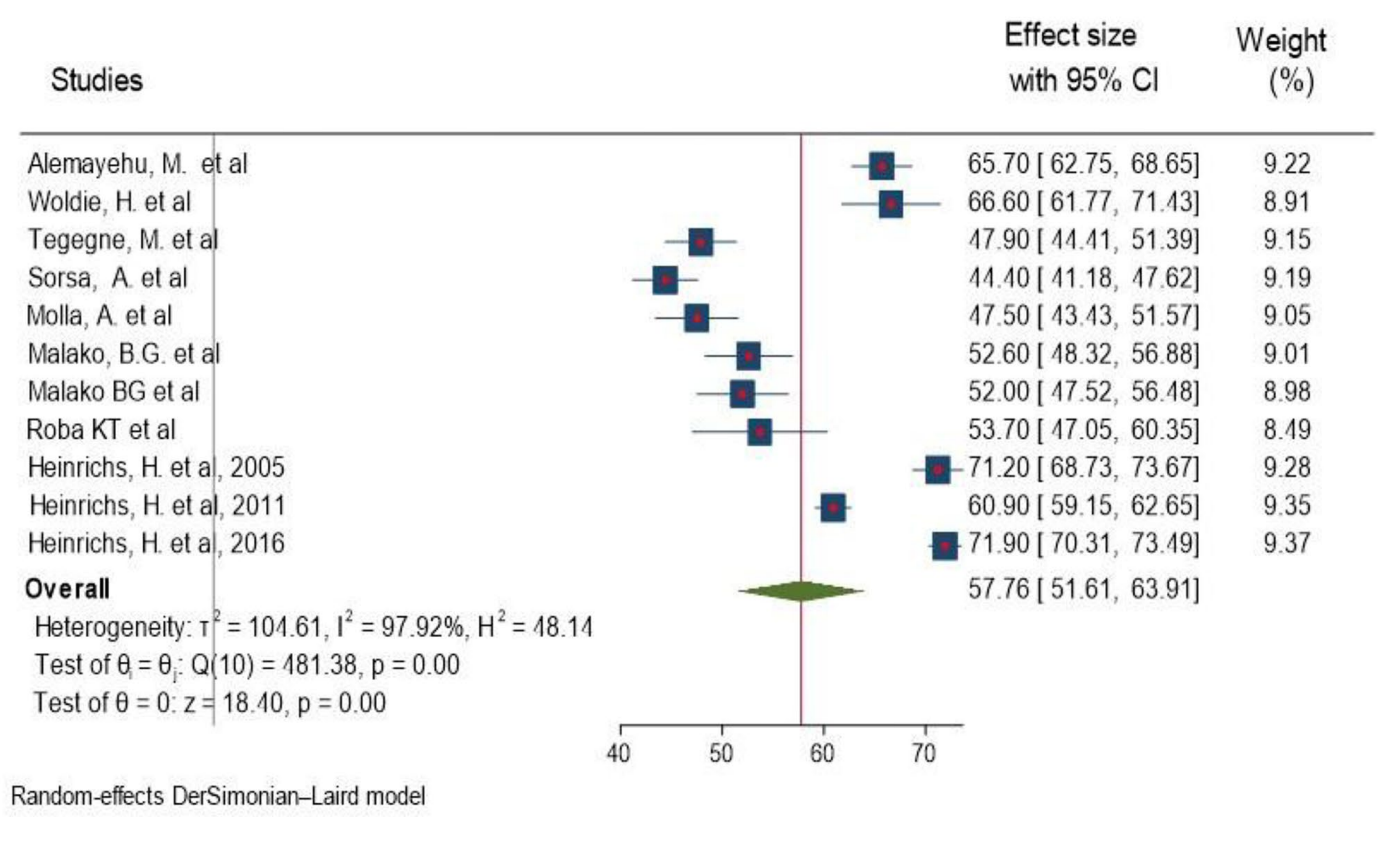
Forest plot of the included studies showing the pooled prevalence of anemia among children aged 6–23 months in Ethiopia, 2023

As described in the study characteristic section of this paper, study conducted by Heinrichs et al. splited as 2005, 2011 and 2016. Study conducted by Gebrehaweria Gebremeskel M. et al. (EDHS 2016) had small sample size as compared to study done by Heinrichs, H. et al. (EDHS 2016) so that study conducted by Gebrehaweria Gebremeskel M et al. was not considered in the pooled prevalence analysis. Most of the studies (n = 10) had reported the prevalence of anemia [13–20, 30, 31]. The random-effects model analysis from those studies revealed that, the pooled prevalence of anemia in Ethiopia was found to be 57.76% (95%CI; 51.61–63.91; I^2^ = 97.192%; p < 0.001) (Fig. 2). I^2^ (97.81%) indicated high heterogeneity. Galbraith plot did not show any significant outlier study (Supplementary Fig. 1).

### Subgroup analysis

The subgroup analysis was done through stratification by region. Based on this, the prevalence of anemia among the study participant was found to be 57% in Amhara, 69.07% in EDHS, 56.88% in SNNPE, 47.88% in Oromia regions (Supplementary Fig. 2).

### Sensitivity analysis

We employed a leave-one-out sensitivity analysis to identify the potential source of heterogeneity in the analysis of the prevalence of anemia with in the study population in Ethiopia. The results of this sensitivity analysis showed that our findings were not dependent on a single study. The estimated pooled prevalence of anemia varied between 56.31(95% CI, 50.33–62.29) [15] and 59.14 (95% CI, 52.00-66.28) [18] after deletion of a single study (Supplementary Fig. 3).

### Publication bias

**Fig. 3:**
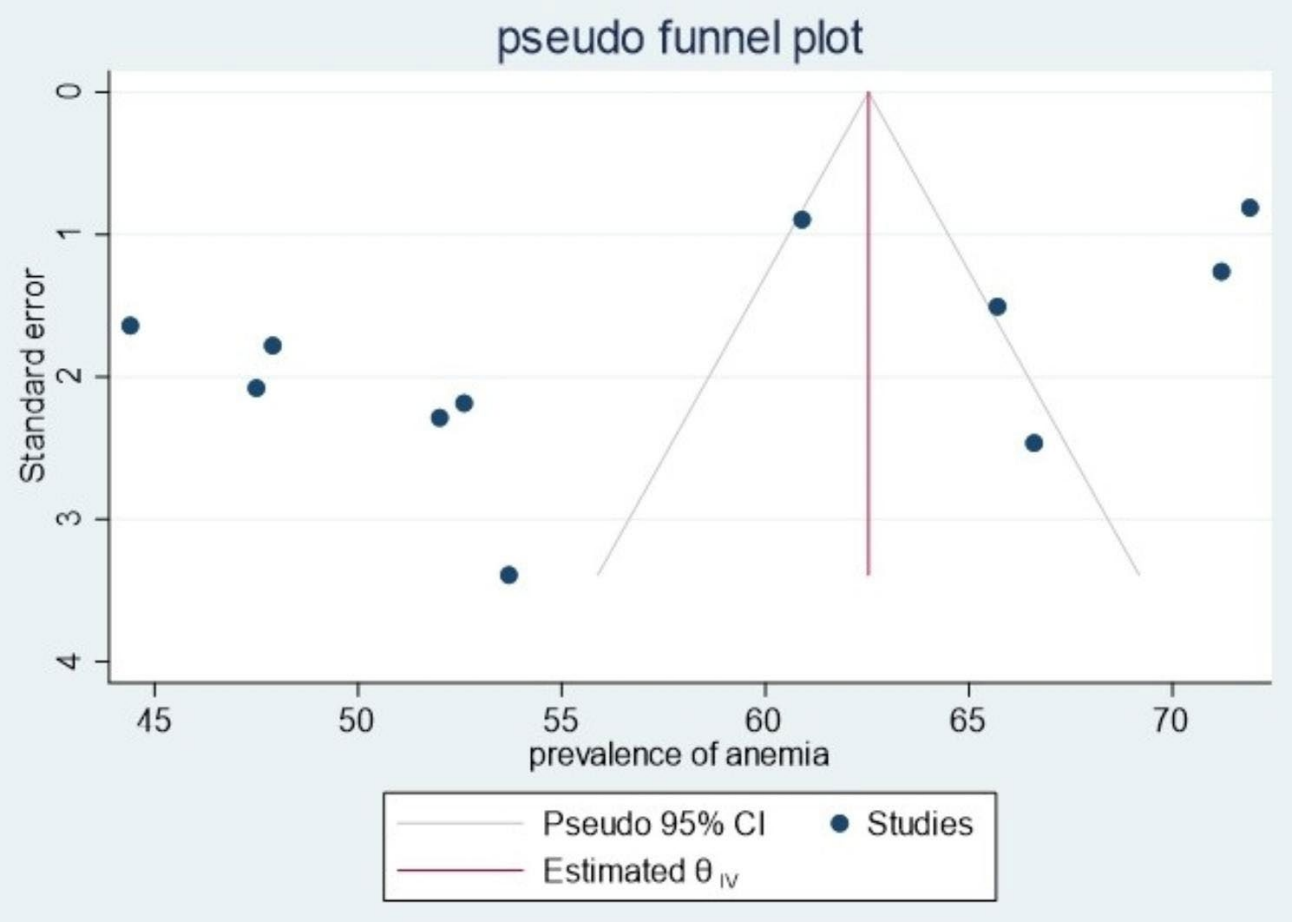
Funnel plot showing no evidence of publication bias estimating the prevalence of anemia in children aged 6–23 months in Ethiopia, 2023

We had also checked publication bias using a funnel plot. It did not show any obviously observed publication bias (Fig. 3). Egger’s regression and begg test p-value were 0.11 and 0.44, respectively, which also indicated the absence of publication bias.

### Factors associated with anemia

All of the included [13–20] except two studies [30, 31] reported at least one factor associated with anemia among children aged 6–23 months in Ethiopia. Even though studies reported that being male, being underweight, poor wealth quantile and early complementary feeding as a significant predictor of anemia in children aged 6–23 months of age; the pooled effect size of these factors did not show any association with anemia. The pooled estimate of having history of diarrhea, being stunted, poor dietary diversification, living in food insecure house hold and being aged between 6 and 11 months were significant predictors of anemia in children 6–23 months of age. For further information, the significant variables were discussed in detail here in the coming sub-topics.

### History of diarrhea

**Fig. 4:**
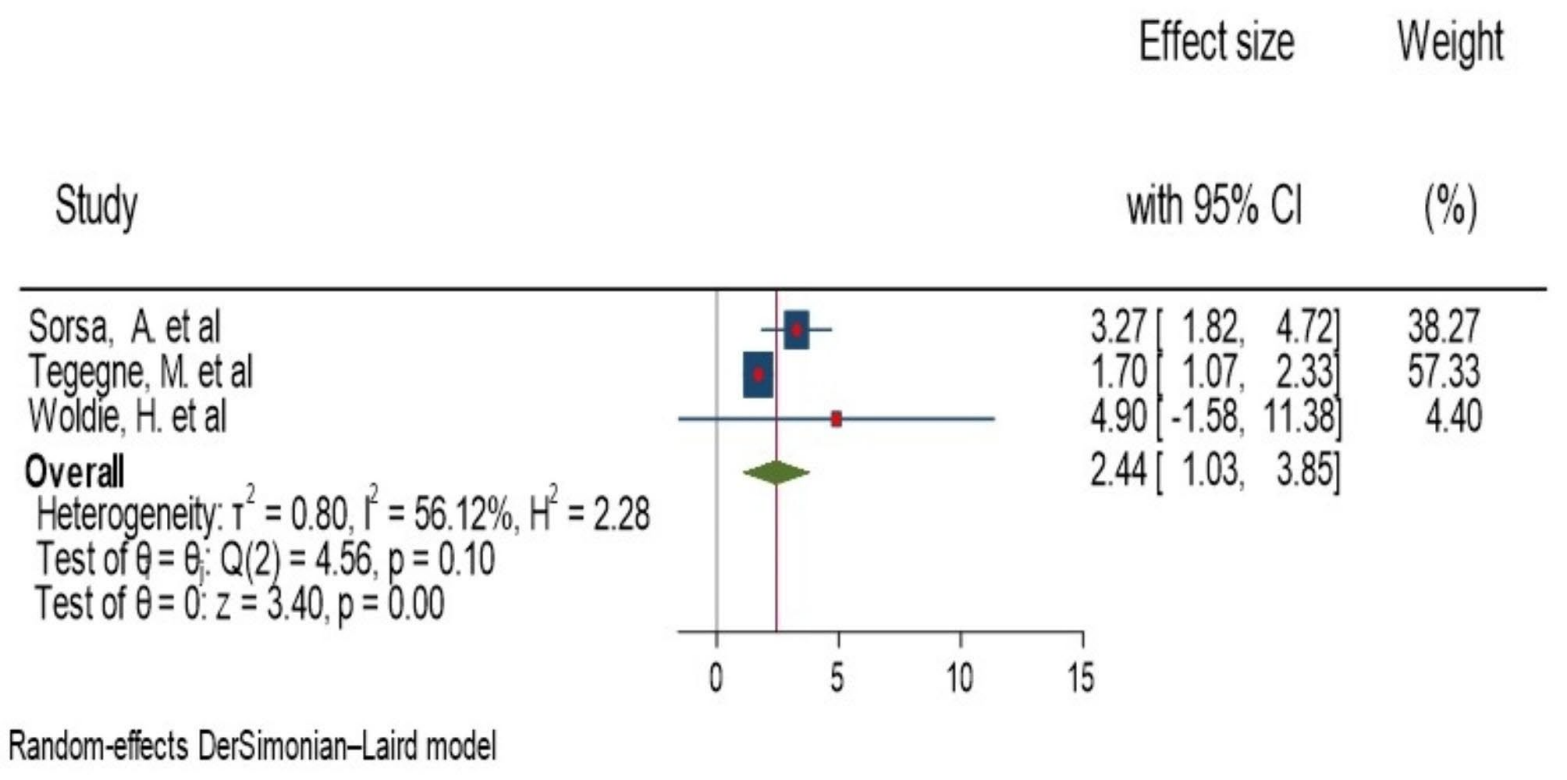
forest plot showing the pooled AOR for association of diarrhea and anemia among children aged 6–23 month

Three studies found significant association between diarrhea and anemia. Of these, the highest and lowest risk factors for children aged 6–23 month with diarrhea were AOR = 4.9 (1.63, 4.9) Woldie, H. et al. [20] and AOR = 1.7(1.18, 2.44) Tegegne, M. et al. [19], respectively, compared to those who had no diarrhea. The forest plot combining result of three studies showed that the overall estimate of AOR of history of diarrhea was 2.44 (95%CI: 1.03–3.85); I^2^ = 56.12%; P = 0.001). I^2^ and P-value showed moderate heterogeneity (Fig. 4).

Regarding to publication bias, the funnel plot analysis showed symmetrical distribution (Supplementary Fig. 4). The Galbraith plot also did not show any outlier (Supplementary Fig. 5). During the Egger’s regression and begg test, the p-value was 0.17 and 1.00, respectively, which indicated the absence of publication bias. Additionally, we employed a leave-one-out sensitivity analysis to identify the potential source of heterogeneity in the analysis of the pooled estimate of history of diarrhea as a risk factor for anemia. The results of this sensitivity analysis showed that pooled effect size was not dependent on a single study. The pooled estimate of history of diarrhea varied between 1.73(95%CI, 1.10–2.36) and 3.35(95%CI, 1.93–4.77) after deletion of a single study (Supplementary Fig. 6).

### Children with stunting

**Fig. 5:**
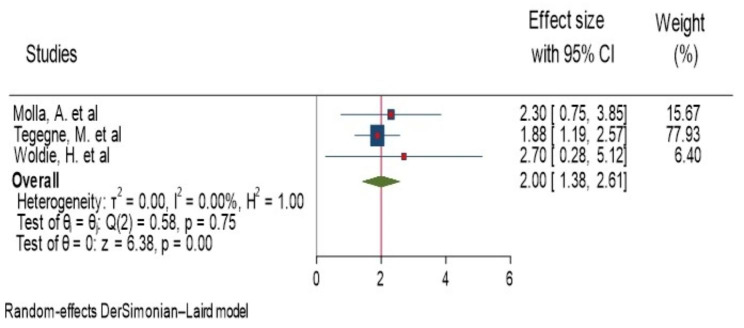
Forest plot showing the pooled AOR of association between stunting and anemia among children aged 6–23 month in Ethiopia, 2023

A total of three studies were reported a significant association between stunting and anemia [17, 19, 20]. Among the significant studies, the highest and lowest risk factors for anemia, in children with stunting were AOR = 2.7 (1.2, 6.05) Woldie, H. et al. [20] and AOR = 1.88(1.31, 2. 7), Tegegne, M. et al [19], respectively, compared to those who had no stunting. The forest plot result of three studies showed that the overall estimate of AOR of stunting was 2.00 (95%CI: 1.38–2.61); I^2^ = 0.0%; P = 0.001). I^2^ and P-value showed homogeneity of the studies (Fig. 5). Regarding to publication of bias, the funnel plot analysis showed symmetrical distribution (Supplementary Fig. 7). The Galbraith plot also did not show any outlier (Supplementary Fig. 8). During the Egger’s regression test, the p-value was 0.45, which indicated the absence of publication bias. The begg test also support absence of publication bias, p-value = 0.30.

### House hold food insecurity

**Fig. 6:**
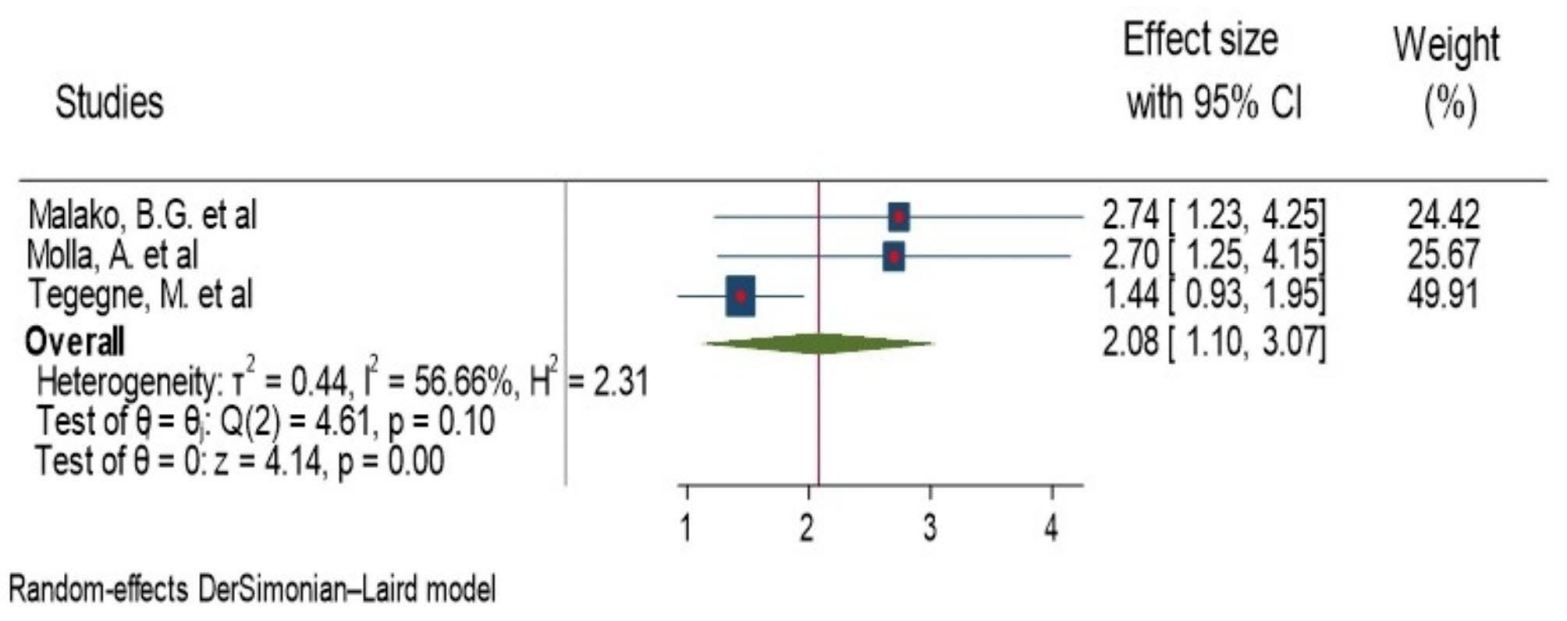
Forest plot showing the pooled AOR for association of house hold food insecurity and anemia among children aged 6–23 month in Ethiopia, 2023

Malako, B.G. et al., Molla et al., and Tegegne, M et al. were reported a significant association of house hold food insecurity and anemia in children aged 6–23 months of age [16, 17, 19]. Among the significant studies, the highest and lowest risk factors for anemia in children living in food insecure house hold were AOR = 2.74 (1.62, 4.65), Malako, B.G. et al. [16] and AOR = 1.44(1.01, 2. 04), Tegegne, M. et al [19], respectively, compared to their counter parts. The heterogeneity test for house hold food insecurity, the combining result of three studies forest plot revealed that the overall estimate of AOR was 2.08 (95%CI: 1.10–3.07); I^2^ = 0.0%; P = 0.001) (Fig. 6). I^2^ and P-value showed homogeneity of the studies. Regarding to publication bias, the funnel plot analysis showed symmetrical distribution (Supplementary Fig. 9). The Galbraith plot also did not show any outlier (Supplementary Fig. 10). During the Egger’s regression and begg test p-value were 0.03 and 1.00, respectively; which indicated the presence of publication bias. Thus, we employed a leave-one-out sensitivity analysis to identify the possible source of bias in the analysis of the pooled estimate of house hold food insecurity as a risk factor for anemia. The results of this sensitivity analysis disclosed that our findings were depend on a single study, Tegegne, M. et al [19] (Supplementary Fig. 11). Our pooled estimate of house hold food insecurity varied between 1.88 (95%CI, 0.7–3.06) and 2.72(95%CI, 1.67–3.77) after deletion of a single study.

#### Dietary diversity

**Fig. 7:**
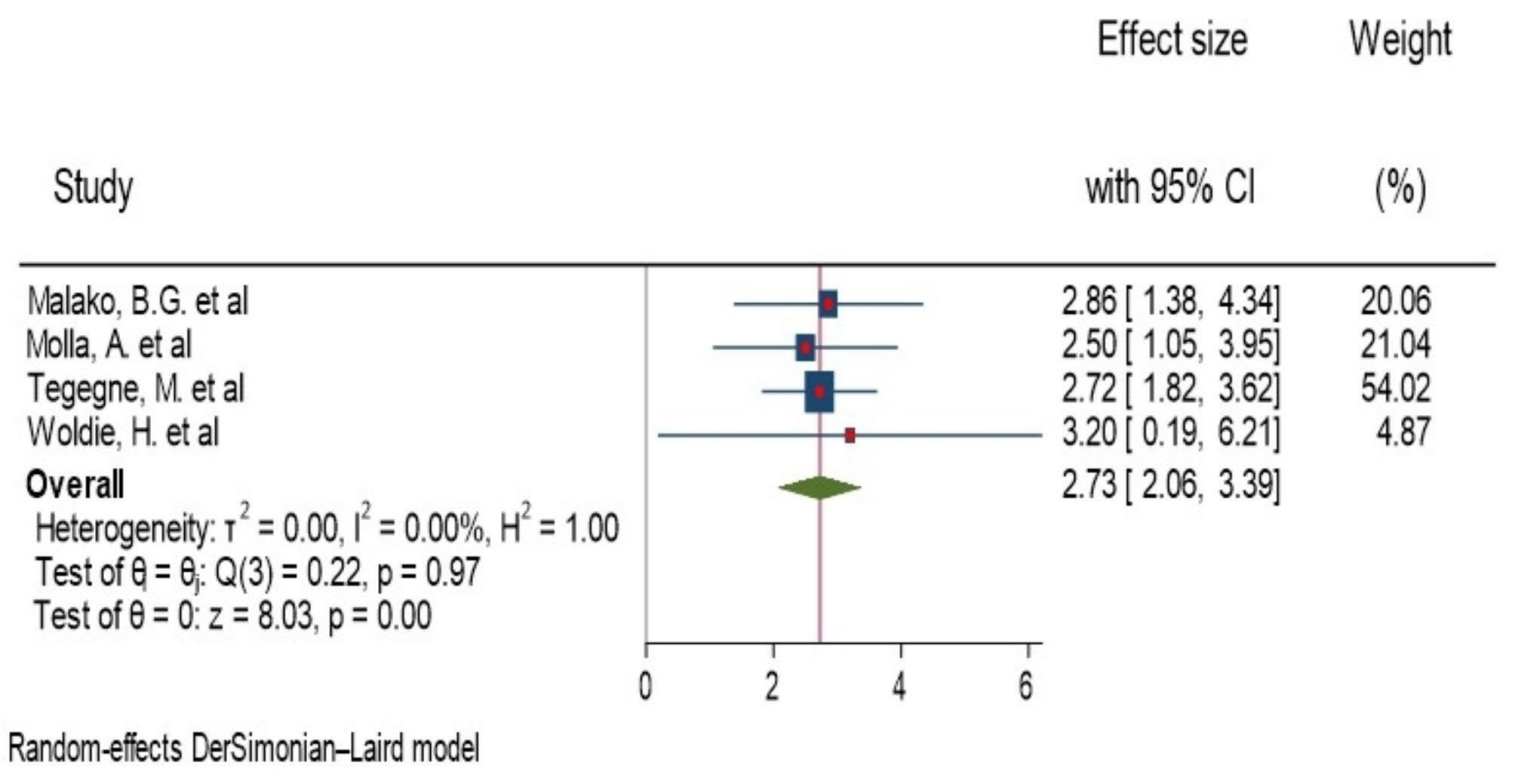
Forest plot showing the pooled AOR for association of dietary diversity and anemia among children aged 6–23 month in Ethiopia, 2023

Malako, B.G. et al., Molla et al., Woldie, H. et al. and Tegegne, M. et al. were stated a significant association of dietary diversity and anemia in children aged 6–23 months of age [16, 17, 19, 20]. Among the significant studies, the highest and lowest risk factors were AOR = 3.2 (1.35, 7.38), Woldie, H. et al. [20] and AOR = 1.4(1.03, 1.92), Menaseb Gebrehaweria, et al. [14], respectively, compared to those who took diversified food (Fig. 7). The heterogeneity test for dietary diversity, the combining result of three studies forest plot presented the overall estimate of AOR was 2.73 (95%CI: 2.06–3.39); I^2^ = 0.0%; P = 0.001) (Fig. 7). I^2^ and P-value showed homogeneity. Regarding to publication of bias, the funnel plot analysis showed symmetrical distribution (Supplementary Fig. 12). The Galbraith plot also did not show any outlier (Supplementary Fig. 13). During the Egger’s regression test, the p-value was 0.81, which indicated the absence of publication bias.

### Age

**Fig. 8:**
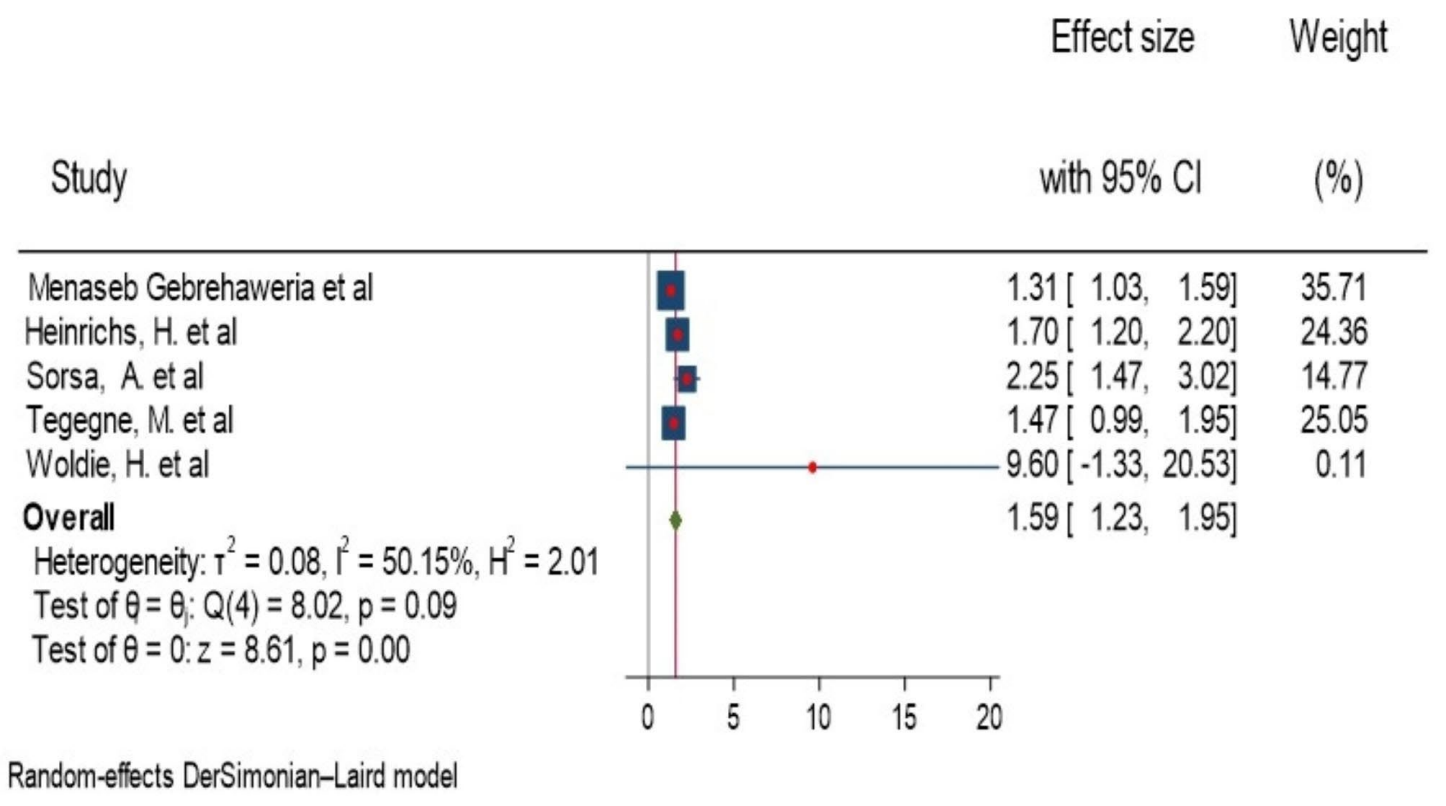
Forest plot showing the pooled AOR for association of age and anemia among children aged 6–23 month in Ethiopia, 2023

Gebrehaweria Gebremeskel M et al., et al., Woldie, H. et al., Sorsa, A. et al., Heinrichs, H. et al. and Tegegne, M et al. were described a significant association of age 6–23 months and anemia in children aged 6–23 months of age [14, 15, 18–20]. The highest and lowest risk factors for children aged 6–11 months were AOR = 9.6 (3.61, 25.47), Woldie, H. et al. [20]and AOR = 1.31(1.07, 1.63), Gebrehaweria Gebremeskel M et al [14], respectively, compared to those whose age ranged from 18 to 23 months among the significant studies (Fig. 8). The heterogeneity test for the combining result of three studies forest plot showed that the overall estimate of AOR for children aged 6–11 months was 1.59 (95%CI: 1.23–1.95); I^2^ = 50.15%; P = 0.001). I-squared and P-value showed heterogeneity of the studies (Fig. 8). Regarding to publication of bias, the funnel plot analysis showed asymmetrical distribution (Supplementary Fig. 14). The Galbraith plot also showed an outlier (Supplementary Fig. 15). During the Egger’s regression test, the p-value was 0.01, which indicated the presence of publication bias. Thus, we employed a leave-one-out sensitivity analysis to identify the potential source of heterogeneity in the analysis of the pooled estimate of 6-11months of age as a risk factor for anemia. The results of this sensitivity analysis showed that our findings were depend on single study (Supplementary Fig. 16). Our pooled estimate of age 6–11 months varied between 1.45(95%CI, 1.17–1.74) and 1.75(95%CI, 1.3–2.2) after deletion of a single study. Then, subgroup analysis was done using study setting. The pooled estimate of AOR for community-based study was 1.75 (95%CI, 1.3–2.2), I^2^ = 37.11% and p = 0.001 and AOR for health institution was 1.31 (95%CI, 1.03–1.59) (Fig. 8).

## Discussion

This systematic and meta-analysis study included 10 studies to assess the pooled prevalence of anemia and associated factors among children 6–23 months of age in Ethiopia which were published from 2015 to 2022. As far as our knowledge is concerned, this systematic and meta-analysis study is the first of its kind in Ethiopia among children aged 6–23 months.

In this study, the pooled prevalence of anemia among children aged 6–23 month was 57.76% which is in the range of WHO severe public health problem [32]. The pooled prevalence is much higher than a previous study done in China [33] and Romania [34]. The discrepancy might be due to socio-economic (absence of iron supplementation in Ethiopia but in China,Ying Yang Bao program which is a free government micronutrient supplementation program that has been in operation since 2009 to improve the health of children in China’s disadvantaged rural communities [35]. High parasitic infestation and inappropriate complementary feeding initiation (early cereal based feeding prolonged milk feeding) were also the possible contributors of discrepancy [10]. A previous study in Latin America and the Caribbean countries also reported that the prevalence of anemia is higher in preschool children than school age children [22]. Similarly, a higher proportion of anemia was also reported in children 1–2 years of age in United States of America [36]. Additionally, the prevalence of anemia is also higher than the previous systematic and meta-analysis study among children aged 6–59 months done in Ethiopia [37]. This discrepancy might be due to growing tissues need an absolute increase in iron content to maintain an iron repletion state, young children’s blood volume must expand, their muscle and tissue mass must rise, and their neurodevelopment and brain development are at a critical stage compared to old child. In addition, iron requirement decreased at the age of third year secondary to decreased growth velocity [10]. This implies that children in early childhood period requires anemia screening as early as possible and the key population, children under 2 years old need to be considered to combat anemia. Furthermore, low amounts of important minerals such as iron, zinc, and folate, as well as vitamin A and B12 in the anemic mother’s breast milk, may affect the hemoglobin level of the breastfeeding child [38]. On the other side, the prevalence of anemia in the population of interest is lower than the previous study, standardized demographics health survey of multiple countries report of anemia (70%) [39]. The discrepancy might be due to methodology including sampling and quality of sample collection in demographic health surveys. Another study report in Myanmar reported that 77.2% of the children aged 6–23 month had anemia which is higher than this study finding [40]. Genetic, ethnic, and geographical lifestyle factors may all interact and can contribute for the discrepancy.

Having history of diarrhea, being stunted, living in food insecure house hold, using less dietary diversity and children aged 6–11 monthswere significantly associated with anemia prevalence.

The odds of anemia among children having history of diarrhea were 2.44 times more likely to develop anemia than children not having diarrhea. A previous studies also reported a similar finding [41]. In normal physiology, iron is absorbed in the proximal doudneum. The increased peristalisis of gastro intestinal system during diarrhea may reduce nutrient absorbtion including iron. Iron distribution regulation functions as an innate defense response against invading microorganisms may also contribute for iron deficiency related anemia. Even in the absence of infection, certain aspects of human iron metabolism guarantee that pathogenic microbes have little access to iron. First, in humans, the majority of iron is sequestered intracellularly, complexed within hemoglobin within erythrocytes. As a result, several diseases have evolved ways to free hemoglobin by lysing erythrocytes and extracting iron from heme. Hemolytic pathogens, on the other hand, must contend with haptoglobin and hemopexin, two host glycoproteins that scavenge freed hemoglobin and heme, respectively [42, 43]. The other hypothesis for anemia secondary to infection is linked to effects of various cytokine mediators of inflammation, such as tumor necrosis factor (TNF), interleukin (IL-1 and IL-6), and the interferons (IFNs)-IFNγ. Neocytolysis (selective hemolysis of the youngest red cells) appears to be a consequence of relative or absolute erythropoietin deficiency. Although mediators of inflammation including cytokines causes anemia secondary to infection, hepcidin is the most significant mediator in the pathogenesis of anemia. It promotes macrophage iron retention by causing internalization of the iron export protein, ferroprotein.

Furthermore, those stunted children age 6–23 months of age were also 2.00 times more likely to be anemic than their counter part. The finding was supported by previous studies [44, 45]. Since, all of the studies included in this study were cross-sectional, they did not show the real causal relationship between anemia and stunting. However, the interrelationships between diet and anemia in young infants may be explained by iron metabolism and the innate immunological response to infection. Iron is an important but harmful component in the human body due to its ability to exist in one of two oxidation forms, ferrous (Fe2+) or ferric (Fe3+). In the ferrous state, iron serves as an electron donor, while in the ferric state, it works as an electron acceptor. Iron’s redox potential can produce reactive oxygen species, which can then produce free radicals that damage lipids, DNA, and protein, especially under conditions of iron excess [46]. Another hypothesis, in low-resource settings, where newborn iron stores may be suboptimal and there is reduced access to food including iron-rich foods which may leads to stunting [47]. In addition, intrauterine growth restriction and preterm delivery, both of which contribute to LBW and small size at birth [47]. Those neonates born prematurely have a tendency to low iron stores and short stature [10, 47, 48].

Similarly, food-insecure children were 2.08 times more likely to develop anemia than secured once. The Food and Agriculture Organization [49] definition of household food insecurity has two broad components: insufficient access to a nutritionally adequate and safe food supply and underutilization of these foods by household members. The access part comprises three main domains: “anxiety and uncertainty about household food supply, inadequate quality of food, and insufficient food to eat by household members” [50, 51]. The utilization component is affected mostly by nutritional knowledge and beliefs; however, access to healthcare, water, sanitation services, hygiene and childhood illness management are also factors [49]. The negative effects of household food insecurity are: decreased food consumption, which comprises of reduced dietary variety and nutrient intake, and under nutrition of household members including micronutrient deficiencies. Children in food-insecure households typically consume diets high in refined sugars and deficient in minerals including iron which result in anemia [52, 53]. In addition, lack of adequate iron consumption; a diet lacking in micronutrients that may facilitate iron absorption and utilization such as vitamin C, vitamin A, folate, vitamin B-12, and carotenoids and consuming foods high in phytic acid, which may reduce iron absorption [52]. Furthermore, previous research on house hold food insecurity and micronutrient deficits in the United States suggests that diets in food insecure households are lower in iron and other micronutrients and higher in carbohydrate and fat [54]. In addition, food insecurity may lead to anemia due to a lack of nutritious diets with high protein quality, enough micronutrient content and bioavailability, macro minerals, iron, and critical fatty acids in children from food insecure households, which increases the likelihood of childhood anemia [55]. Generally, household members live in food insecure house hold obligated to consume less diversified foods [16].

Children who received less diversified food were more likely to be anemic than those who received diversified one. The finding was supported by studies done in Ghana [56] and China [57]. WHO defined minimum dietary diversity as the proportion of children aged 6–23 months who received foods from at least four out of seven food groups in a 24 h time period [58].

Children whose age 6–11 month were 1.59 times more likely to develop anemia as compared with children whose age 18–23 months. Breast milk has inadequate iron to meet the nutritional iron need of growing infant [59]. Provision breast milk alone coupled with rapid iron depletion beyond six months also increases risk of anemia for younger infant. The main source of iron for young infant is their iron store during intra uterine life. After birth, they are prone for iron deficiency due to the increased postnatal iron required to facilitate rapid growth and an earlier onset of erythropoiesis, which occurs 1-3months earlier in preterm than term infants [12]. Unless appropriate complementary feeding is initiated and iron supplemented for preterm and risk term, the existing iron store become gradually depleted and fail to meet the demand. Iron supplementation is recommended after 1 months of age in preterm and 4 month of age for term infants, especially those infants live in anemia prevalent area [10, 60]. This study finding was supported by previous studies [33, 61, 62]. This might be due to increased nutrition demand for growth at late infant(6–11 months) age [63], maternal anemia, early cord clamping prenatal iron store depletion and poor knowledge of care givers in complementary feeding composition (iron poor food) and complementary feeding initiation (being late or early) [64–66]. American academic of pediatrics also reason out that the amount of iron lost was added to the amounts of iron necessary for increased blood volume, increased tissue mass, and storage iron throughout the age group of 6–12 months, mostly from sloughed epithelial cells from the skin and the intestinal and urinary tracts [67]. Being born from mother with maternal anemia may exposed young infants early for anemia due to low iron store [10]. This implies that anemia screening and iron reach food or supplementation requirement with in the first year of life.

### Limitation of study

Since, all of the included studies were cross-sectional, the result cannot show the real cause-effect relationship between anemia and identified factors. In Ethiopia, there is variation in demography (low land and high land) and living standard (pastoralists and agrarian). These may contribute for variety in food composition and accessibility. Since, most of the included studies except EDHS were from only three productive administrative regions, the pooled prevalence of anemia may be under estimated in Ethiopia. As a result, it is difficult to represent the true effect within the country because research from more than half of the areas were scarce. The use of varied data collection tools in the included research may have an impact on generalizing the findings. Furthermore, the study’s pooling of prevalence and odds ratio despite significant variability is one of its limitations. Even though no language exist other than English is available for health and health related article publications in Ethiopia, restriction of searching to English language may be the limitation of this study.

## Conclusion

According to this study report, the prevalence of anemia is high as compared to the world health organization anemia grading. Diarrhea, stunting, house hold food insecurity, dietary diversity, and age were the predictors of anemia. Anemia has a tendency to hinder the children capacity for learning, psychomotor development, cognitive development, behavioral development, physical growth, and raising the likelihood of illness including high risk of infection and work productivity later in life. To halt this effect of anemia, strengthening diarrhea reduction program, securing household food insecurity, preventing stunting, giving special attention for infants and encouraging food diversification are important.

### Recommendation

An interventional random control trial is needed to determine cause-effect relationship of anemia and predictors. Since Most iron-rich foods are available in Ethiopia and iron deficiency is most cause of anemia in children, effectiveness of nutritional education interventions trial is recommended.

Strengthening nutritional intervention including appropriate complementary feeding composition and initiation, anemia screening during infancy period, nutritional education programs including dietary diversification, encouraging hygiene and sanitation practices, prevention of anemia in pregnant women and launching iron supplementation program based on World Health Organization guideline could help to prevent childhood anemia in Ethiopia and possibly reduce the prevalence. In addition, infants (preterm and risk term at one month and four months of birth, respectively) require a special consideration for iron supplementation program to prevent anemia. The health care providers need to screen children age 6–23 months for anemia who presents with diarrhea.

Furthermore, care givers need to be encouraged to include iron reach foods in the complementary food preparation like lentil, soybean, spinach, meat etc. In the same way, they need to be aware about hinders of iron absorption like tea which contain tannin.

Lastly, since anemia has no single cause, it requires the involvement individual to community at large to reduce the burden and its sequels. This can be achieved through strengthening frequent communication using the most accessible media to create awareness on importance of food diversity, infant and young children feeding, producing nutrient rich agricultural products, health seeking behaviors (early detection of chronic infection) of the community and working on food insecurity in collaboration with health care and agricultural stakeholders targeted to anemia reduction.

### Implication to policy

Evidences suggest that the prevalence of anemia is very high among children aged 6–23 months. The finding of this study, anemia prevalence 57.76%, strengthen the previous studies report on prevalence of anemia in children aged 6–23 months. If children aged 6–23 months are harmed by nutrition-related illness; technological innovation, work productivity, discovery, and societal advancement cannot be expected. Even though, 2 mg per day after 1 month of age for preterm and 1 mg per day after 4 months for term neonate iron supplementation is recommended by American Academy of Pediatrics which requires commitment of programmers working on the areas and the government to launch iron supplementation program and mobilize resource for preterm and risk term neonates. all alternative intervention need to be implemented as soon as feasible to prevent anemia in children. This could be accomplished through strengthening training on infant and young children feeding practice for front line health care workers and health education for care givers, launching anemia screening at late infancy age and education on food diversification to the community, education on complimentary feeding composition and timing, conducting a serious of effectiveness of nutritional education interventions and strengthening maternal anemia prevention programs (malaria prevention and iron supplementation). Generally, the following points can be recommended as priority action areas to reduce the prevalence of anemia and its consequence: Conducting a study on effectiveness of nutritional education interventions trials at national level; launching iron supplementation program for all preterm neonate and risk term neonate (like born from mother with anemic, in food insecure household, with diabetic) and due to multifactorial nature of anemia causes, launching anemia screening program at late infancy period may reduce the long-term impact of anemia. This could be integrated with measle vaccination. The implementation of these anemia reduction program may require commitment of the government, donors, high resource mobilization and inter sectoral collaboration like health sector and agricultural sectors. However, anemia prevention and control programs may face several challenges from the side of policy maker(political priority, poor coordination of resources to support multisectoral response and less financial commitment), resource-human and financial (challenge of integration of anemia control program into existing programs, poor awareness of need for anemia prevention and control) and directly the beneficiary-care givers (lack of education and information about anemia prevention, restricted financial access to participate in anemia control and prevention strategies (iron-rich food sources, iron supplements, health services, etc.), poor awareness of benefits of interventions and poor compliance with iron supplementation).

## Implication to further research

The meta-analysis found that the prevalence of anemia was quite high among children aged 6–23 months and the primary reasons for anemia burden were identified. However, the included studies were too diverse, and cross-sectional research also failed to demonstrate a temporal association between anemia and its factors. In addition, the measurements of exposure and their effect on the outcome of this study were made at the same time. It is not easy to assess the reasons for existing directions of associations. The study finding may also be prone for mediator and confounding variables effect. As a result, there is a potential for reverse causality of the identified factors. So, prospective studies assessing the predictors and the outcomes of anemia in children 6–23 months such as effectiveness of nutritional education interventions trials are required by stratifying the possible confounding and mediator variables.

### Electronic supplementary material

Below is the link to the electronic supplementary material.

Supplementary Material 1

## Data Availability

All relevant data are available within the paper. In addition, the corresponding author will provide anonymized data upon reasonable request from researchers who require more information.
