# Supplementary material for: Prevalence of anemia and its associated factors among children aged 6–23 months, in Ethiopia: a systematic review and meta analysis
Source: BMC Public Health. 2023 Dec 2;23:2398. doi: 10.1186/s12889-023-17330-y (PMC10693156; doi:10.1186/s12889-023-17330-y)
Supplement: Supplementary file 1 — Supplementary Material 1 [file 12889_2023_17330_MOESM1_ESM.docx]

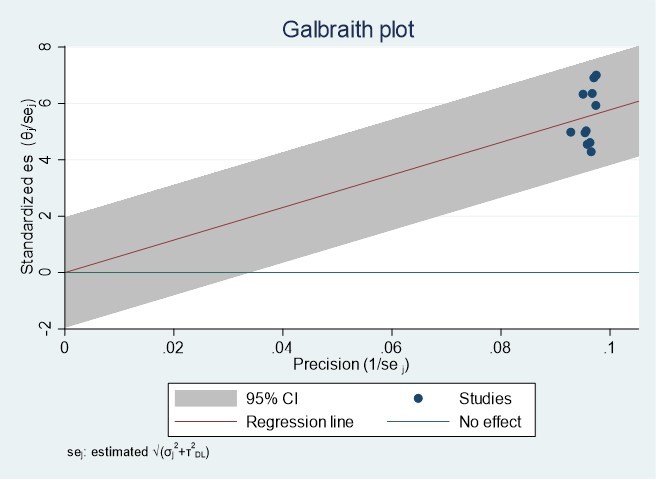


Supplementary figure 1 Galbraith plot showing no evidence of presence of outlier for estimating the prevalence of anemia in children aged 6-23 months in Ethiopia, 2023.


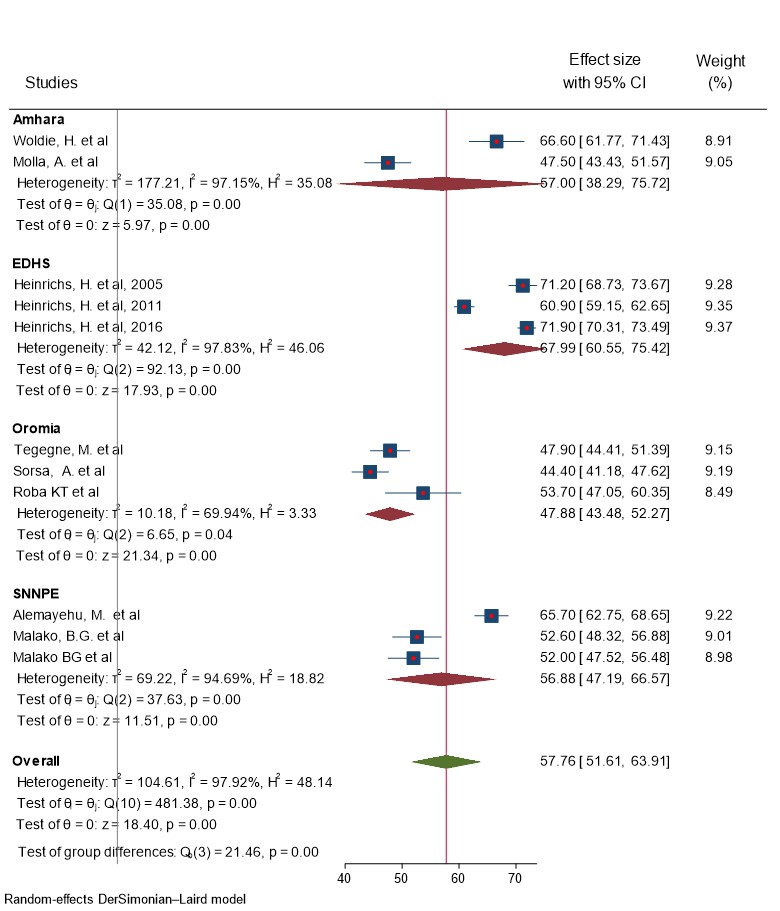


Supplementary figure 2 Forest plot of the included studies showing subgroup pooled prevalence of anemia using region among children aged 6-23 months in Ethiopia, 2023.


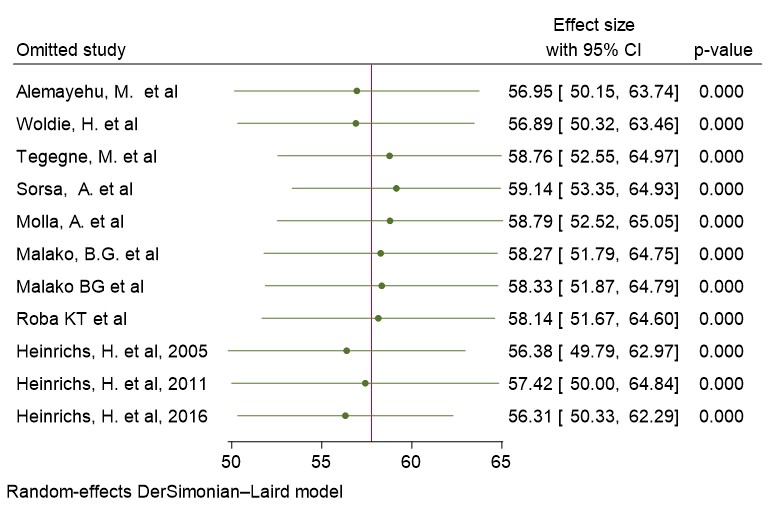


Supplementary figure 3 Forest plot of the included studies showing sensitivity analysis of the included studies on anemia among children aged 6-23 months in Ethiopia, 2023.


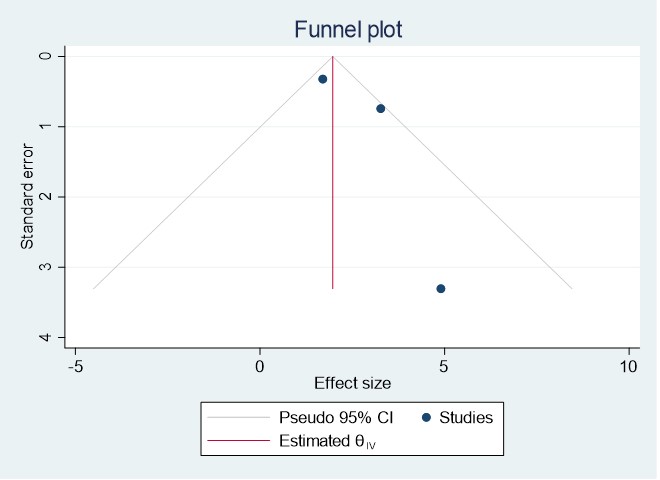


Supplementary figure 4 Funnel plot showing no evidence of publication bias estimating the AOR of the association between diarrhea and anemia in children aged 6-23 months in Ethiopia, 2023.


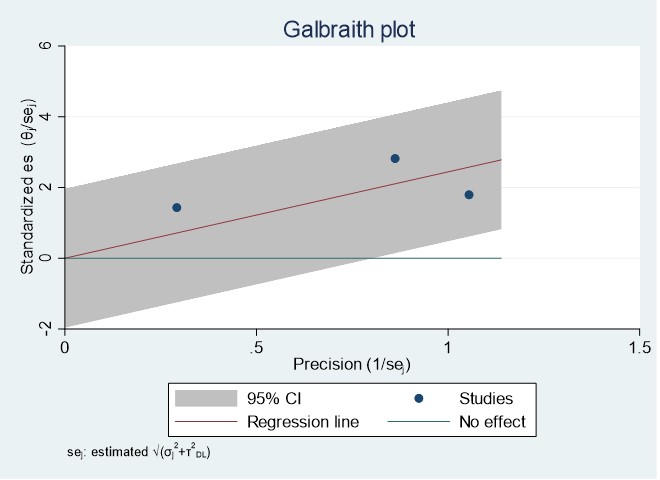


Supplementary figure 5 Galbraith plot showing no evidence of presence of outlier for estimating the prevalence of anemia in children aged 6-23 months in Ethiopia.


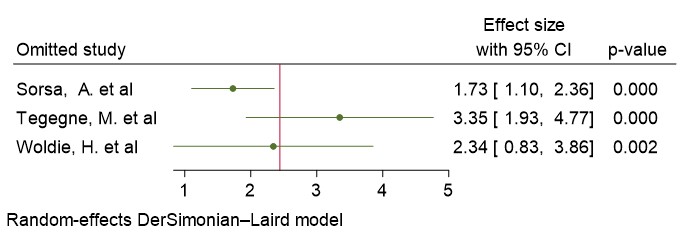


Supplementary figure 6 Forest plot showing sensitivity analysis by omitting one study at time to diagnose one study effect on the pooled AOR of diarrhea and anemia association among children aged 6-23 month in Ethiopia, 2023


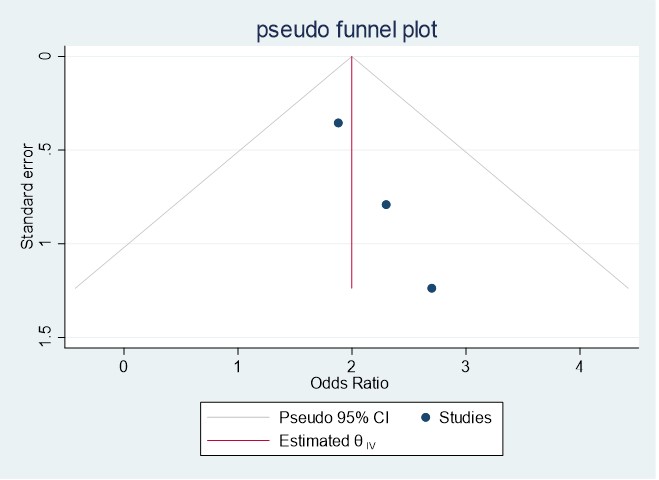


Supplementary figure 7 Funnel plot showing no evidence of publication bias estimating the AOR of the association between stunting and anemia in children aged 6-23 months in Ethiopia, 2023.


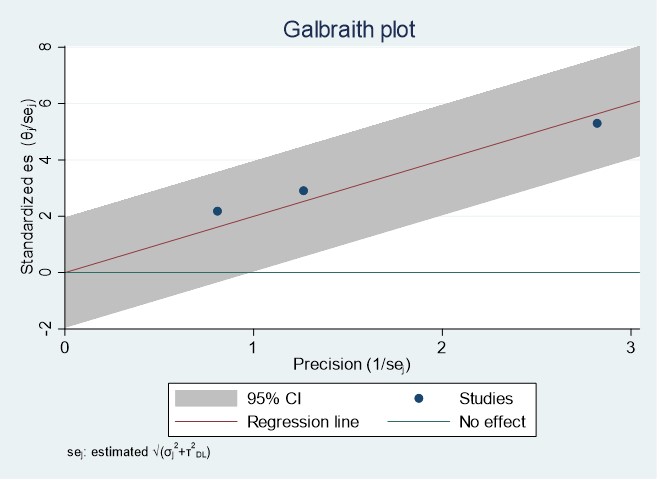


Supplementary figure 8 Galbraith plot showing no evidence of presence of outlier for estimating the prevalence of anemia in children aged 6-23 months in Ethiopia.

**
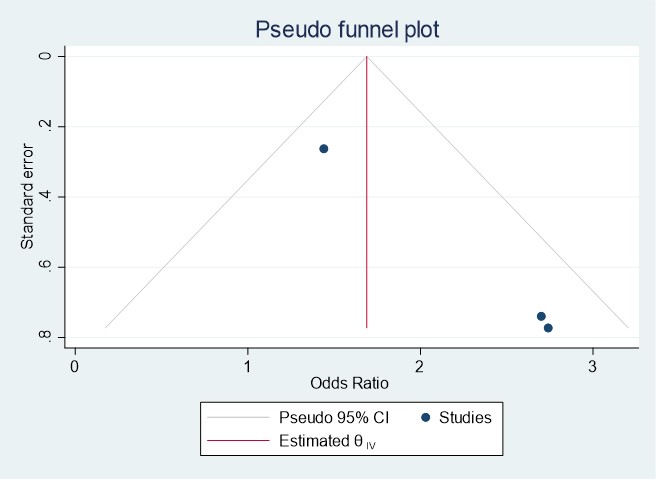
**

Supplementary figure 9 Funnel plot showing no evidence of publication bias estimating the AOR of the association between house hold food insecurity and anemia in children aged 6-23 months in Ethiopia, 2023.


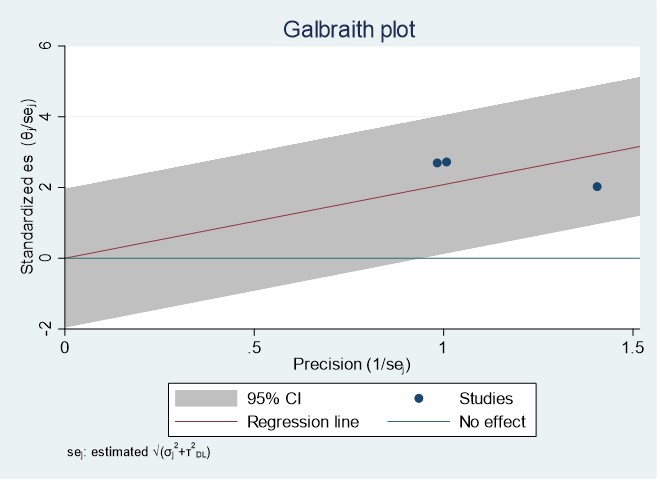
 `

Supplementary figure 10 Galbraith plot showing no evidence of presence of outlier for estimating the prevalence of anemia in children aged 6-23 months in Ethiopia, 2023.


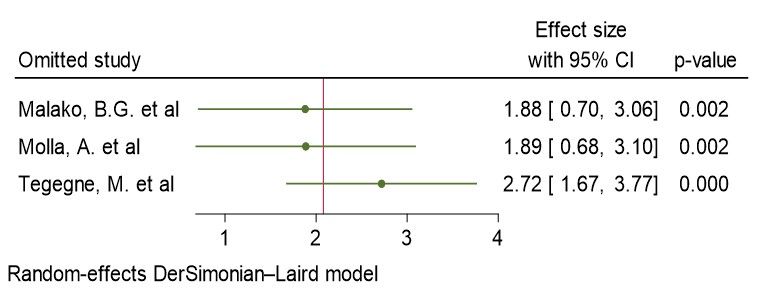
 Supplementary figure 11 Forest plot showing sensitivity analysis by omitting one study at time to diagnose one study effect on the pooled AOR of house hold food insecurity and anemia association among children aged 6-23 month in Ethiopia, 2023


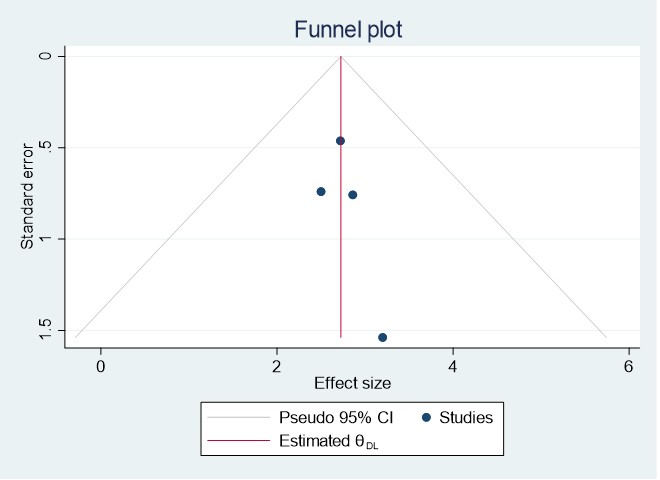


Supplementary figure 12 Funnel plot showing no evidence of publication bias estimating the AOR of the association between dietary diversity and anemia in children aged 6-23 months in Ethiopia, 2023.


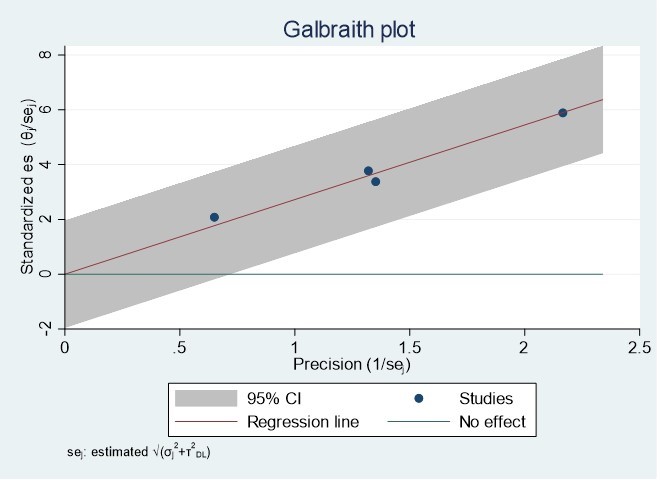


Supplementary figure 13 Galbraith plot showing no evidence of presence of outlier for estimating the prevalence of anemia in children aged 6-23 months in Ethiopia.


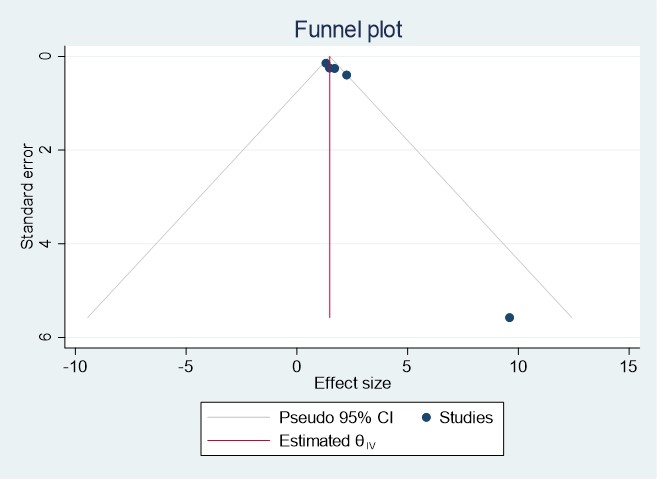


Supplementary figure 14 Funnel plot showing no evidence of publication bias estimating the AOR of the association between age and anemia in children aged 6-23 months in Ethiopia, 2023.


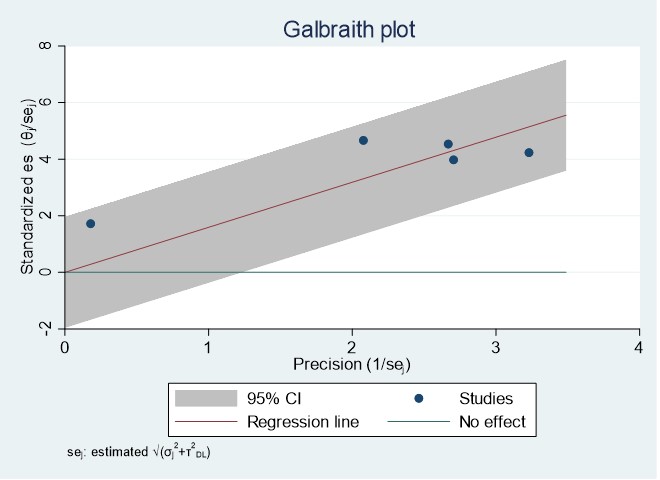


Supplementary figure 15 Galbraith plot showing no evidence of presence of outlier for estimating the prevalence of anemia in children aged 6-23 months in Ethiopia, 2023.


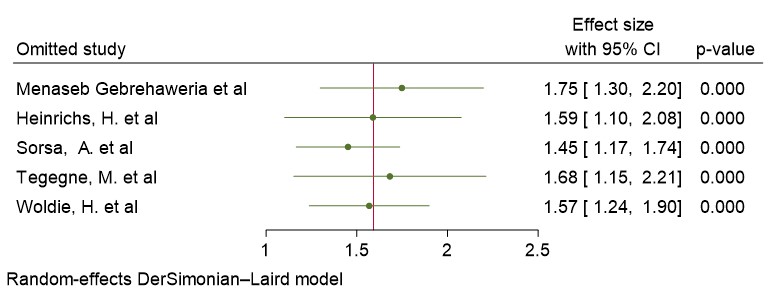


Supplementary figure 16 Forest plot showing sensitivity analysis by omitting one study at time to diagnose one study effect on the pooled AOR of age and anemia association among children aged 6-23 month in Ethiopia, 2023
